# New Drugs, Applances, and Things Medical

**Published:** 1890-02-08

**Authors:** 


### NEW DRUGS, APPLIANCES, AND THINGS MEDICAL.

### GUNN'S "FOOD OF LIFE."

This is a good specimen of well-manufactured oatmeal, and for those who can digest oatmeal we can thoroughly recommend it. We have carefully examined it, both microscopically and with the usual tests. The great fault of most preparations of oats is the presence of hard, indigestible husk and fibre. We have known cases where obstinate constipation was caused by the presence of these, and, until the cause had been searched for and discovered, much doubt and anxiety had arisen. The above preparation, though having a rather fanciful name, may be taken to be a really good food, and free from these objections of husk and fibre. Here we may note that many people cannot digest even good oatmeal, having an idiosyncracy with regard to it. Again, others, when in the North, have heartily enjoyed the porridge for breadfast, but when South, forgetting the accompaniments of the keen, fresh air and regular, and even hard, exercise, have endeavoured to dispose of an average Scotch breakfast allowance. They wonder why they are so flatulent and dyspeptic; it cannot be the porridge, for that suited them so admirably when on the moors or at the Hydropathic. They were so pleased at having discovered a new dish. But it is the porridge which is in fault, and that for two reasons. First, too much is eaten. On returning south the meal should be lessened in proportion to the work done. Second, the cook is often quite ignorant of how to make it. It should be thoroughly boiled over night, and again in the morning. The tough, fibrous nature of the oat prevents the due cooking of the starch cells, and unless these are cracked by heat, they are not digested to any extent. This fibrous character may be largely obviated by careful grinding and milling, thus facilitating the cooking. The specimen under review appears to be very carefully prepared.

### ALEXANDER'S BEEF WINE.

This is stated to be a solution in wine of extract of meat with quinine and other drugs. The product is of a dark sherry colour, smelling distinctly of the extract. It is a fairly palatable production, and may no doubt be of use as a tonic stimulant. Such combinations as these should only be taken under the advice of a medical man. The public is far too fond of drugging itself with tonics and "pick-me-ups." One of the commonest statements made in a consulting room is "Oh! I took So-and-so's tonic, but I did not seem to improve," the reason in nine cases out of ten being that the patient began with a tonic when he ought to have begun with an alterative or other form of medicine.

